# Improving TCM question answering through tree-organized self-reflective retrieval with LLMs

**DOI:** 10.3389/fmed.2026.1752778

**Published:** 2026-03-12

**Authors:** Chang Liu, Ying Chang, Jianmin Li, Yiqian Qu, Yu Li, Lingyong Cao, Shuyuan Lin

**Affiliations:** 1 School of Basic Medical Sciences, Zhejiang Chinese Medical University, Hangzhou, China; 2 Breast Disease Specialist Hospital of Guangdong Provincial Hospital of Chinese Medicine, Guangdong Provincial Hospital of Chinese Medicine, Guangzhou, China; 3 Gancao Doctor Institute of Artificial Intelligence for Chinese Medicine, Zhejiang Chinese Medical University, Hangzhou, China; 4 Faculty of Chinese Medicine, Macau University of Science and Technology, Macau, Macau SAR, China

**Keywords:** artificial intelligence, knowledge graph, large language model, medical dialogue system, Traditional Chinese Medicine

## Abstract

**Background:**

Large language models (LLMs) offer significant potential for intelligent question answering (Q&A) in healthcare, yet traditional knowledge representation methods fail to capture the complex, hierarchical nature of Traditional Chinese Medicine (TCM) knowledge systems. The lack of effective retrieval-augmented generation (RAG) frameworks specifically tailored for TCM’s unique epistemology limits applications.

**Objectives:**

This study aims to evaluate the effectiveness of a novel Tree-Organized Self-Reflective Retrieval (TOSRR) framework in enhancing LLM performance on TCM Q&A tasks through innovative knowledge organization and dynamic self-correction mechanisms.

**Methods:**

We developed a hierarchical knowledge representation system that structures TCM knowledge as subject-predicate-object-text (SPO-T) units within a tree-like architecture, enabling multi-dimensional relationships while preserving semantic context. Our iterative self-reflection mechanism implements dynamic knowledge retrieval and validation across textbook chapters and disciplines. Performance was evaluated using randomly selected questions from the TCM Medical Licensing Examination (MLE) and college Classics Course Exam (CCE), representing both standardized clinical knowledge and classical theory assessment.

**Results:**

When integrated with GPT-4, the TOSRR framework demonstrated a 19.85% improvement in absolute accuracy on the TCM MLE benchmark and increased recall accuracy from 27 to 38% on CCE datasets. Expert manual evaluation revealed substantial enhancements across critical dimensions: safety, consistency, explainability, compliance, and coherence, with a comprehensive improvement of 18.64 points. Retrieval-Augmented Generation Assessment (RAGAs) metrics confirmed the framework’s superior knowledge utilization, retrieval precision, and resistance to information noise compared to standard RAG approaches.

**Conclusion:**

The TOSRR framework enhances LLM performance in TCM knowledge tasks through its hierarchical knowledge representation and self-reflective retrieval approach. And the framework has potential for application in teaching.

## 1 Introduction

Traditional Chinese Medicine (TCM), as a complex medical system characterized by holistic philosophy and personalized diagnosis and treatment, has extended its services to 196 countries and regions worldwide, covering approximately one-third of the global population. TCM demonstrates unique value in chronic disease management, preventive medicine, and public health emergency response, particularly serving as a critical complementary medical resource in underserved regions (1–3). However, the intricate, multi-layered, and implicitly interconnected knowledge system of TCM presents significant challenges in learning and necessitates prolonged training periods for practitioners. This directly contributes to the current shortage of TCM clinical resources.

In recent years, intelligent question-answering systems based on large language models (LLMs) have enabled efficient human-machine interaction through their advanced natural language understanding and generation capabilities, offering novel technological support for the field of TCM (4). By leveraging deep semantic parsing and knowledge reasoning, these systems facilitate the intelligent dissemination of TCM knowledge in a conversational manner, thereby enhancing the efficiency of practitioner training. At the clinical level, such systems can provide real-time decision support, including syndrome differentiation analysis and personalized prescription recommendations, thereby improving the quality and accessibility of TCM services (5, 6).However, current TCM intelligent question-answering systems based on LLMs still face several critical challenges. First, due to the imbalance in the distribution of Chinese and Western medical knowledge in pretraining corpora, these models are prone to comprehension biases and factual hallucinations when handling TCM-related tasks (7, 8). Second, TCM is a highly structured medical paradigm with a rigorous theoretical framework. Its hierarchical method of knowledge organization differs from the unsupervised learning-based knowledge acquisition approach of LLMs, making it difficult for these models to capture long-range dependencies between distinct conceptual structures (9, 10). Finally, LLMs exhibit generative randomness, and their unidirectional modeling mechanism limits their ability to establish bidirectional logical associations or perform reflective and backtracking reasoning for reverse inference (11). These technical limitations may compromise the medical professional capability of models. Therefore, there is an urgent need to explore effective approaches for systematically integrating TCM domain knowledge into LLMs.

The Retrieval-Augmented Generation (RAG) framework provides a viable solution for domain-specific knowledge integration by combining sequence-to-sequence models with external knowledge bases (12). This technology employs embedding models to vectorize queries, retrieves relevant knowledge fragments through approximate nearest neighbor search, and subsequently inputs them into LLMs to generate final responses, significantly enhancing system interpretability and reliability (13). However, conventional RAG frameworks exhibit notable adaptation challenges when applied to TCM question-answering systems (14). The fundamental issue stems from a structural incompatibility between the inherent hierarchical complexity and semantic richness of TCM knowledge systems and existing knowledge representation methods. Specifically, these systems struggle to effectively capture cross-chapter conceptual associations and interdisciplinary knowledge mappings, ultimately manifesting as incomplete knowledge retrieval and logical discontinuities in response generation (7). Research indicates that employing tree-structured representations to capture textual details can substantially improve retrieval-augmented performance (15). Furthermore, incorporating TCM knowledge structures is considered a promising approach to address the inherent limitations of medical LLMs (8).Therefore, this study innovatively proposes the Tree-organized Self-reflective Retrieval (TOSRR) framework. The framework introduces a multi-level knowledge representation approach that combines SPO (Subject-Predicate-Object) triplets with textual content, enabling structured modeling of TCM’s complex knowledge system. Furthermore, the incorporated self-reflective mechanism (12) endows the system with fact-checking capability through dynamic knowledge retrieval and verification processes. An innovative cross-chapter and interdisciplinary knowledge association and integration mechanism is developed to address TCM’s holistic reasoning requirements. The model’s generation results are being demonstrated on a dedicated webpage (16). Our current work makes the following contributions:

1. We propose a knowledge representation framework based on a tree diagram, which constructs a tree-like structure to store the knowledge base for RAG.
2. We introduce a self-reflective mechanism to enhance the precision of knowledge retrieval and generation.
3. We created a specialized multi-dimensional evaluation dataset for TCM, including data from the TCM Medical Licensing Examination (MLE) and Classics Course Exam (CCE). This dataset allows for a comprehensive evaluation of the model’s understanding of TCM foundational knowledge, medical diagnosis, and classical theory, providing a customized benchmark for intelligent TCM Q&A systems.

The purpose of this study is to address the critical issues of insufficient hierarchical knowledge representation, lack of dynamic self-reflection, and absence of domain-specific evaluation in LLMs for TCM question-answering tasks by proposing the TOSRR framework. The research focuses on enhancing the recall and generation accuracy of models in TCM Q&A tasks through the RAG approach. Model training and fine-tuning are not involved in this study.

## 2 Materials and methods

### 2.1 Related works

#### 2.1.1 Researches of LLM in medical field

LLM has been extensively employed in biomedical research for purposes such as diagnosis, treatment, drug recommendation, and medical advice. Med-PaLM and Med-PaLM2 achieve accuracy of 67.2 and 86.5%, respectively, surpassing the “pass” score on the USMLE (17, 18). PMC-LLaMA excels on PubMedQ&A, MedMCQ&A, and USMLE benchmarks (19). DoctorGLM provides a customizable solution for clinical departments (20).

In the domain of Chinese, several large language models have been developed with different design emphases. Huatuo (21), BiomedGPT (22), BianQue, QiLin (23), and ZhongJing (24) primarily focus on general medical dialogue and clinical question answering in Chinese, leveraging diverse training strategies such as preference alignment, multi-turn dialogue modeling, and large-scale medical text integration to enhance interaction quality and domain coverage.

In contrast, models explicitly targeting TCM remain limited. Qibo (25) is fine-tuned on large-scale TCM corpora to support TCM-related tasks, while Hengqin-RA-v1 (26) is designed for TCM-based diagnosis and treatment in rheumatoid arthritis by integrating TCM principles with modern medical information. Given the current scarcity of specialized TCM models, we selected GPT-4 as the baseline model based on its performance on TCMBench (27), a TCM-specific evaluation dataset, where GPT-4 achieved the best results on TCM licensed examination questions.

By dynamically integrating external knowledge bases with generative capabilities, RAG markedly enhances model performance in knowledge-intensive tasks. Compared with model training or traditional fine-tuning methods, RAG possesses several advantages, including low cost, high efficiency (28), explainable generation sources (29), and real-time capability (30). Although TCM MLKG-RAG introduces multi-level retrieval for TCM knowledge, its retrieval process remains largely static and lacks explicit support for fine-grained, reasoning aligned with syndrome differentiation and treatment determination. We propose a self-reflective RAG framework based on a fine-grained, tree-structured knowledge graph, enabling deeper logical inference and closed-loop retrieval–generation–evaluation. This design is intended to fit the cognitive process of expert TCM reasoning and improves interpretability and adaptability.

#### 2.1.2 Researches of TCM knowledge base

The diagnostic processes of Traditional Chinese Medicine (TCM) involve complex reasoning and fuzzy logic (31), drawing upon a wide knowledge base derived from literature and clinical experience (32). To solve a clinical problem of TCM, it is often necessary to make use of knowledge points that span many books and chapters such as Diagnostics, Formulations, and Chinese Pharmacy. KG can organize TCM knowledge in a structured form, retaining the hierarchy and relevance of knowledge (33), and facilitating cross-chapter knowledge inquiry. KGs have been instrumental in developing intelligent inquiry systems, aimed at improving the efficiency and precision of medical queries in TCM (34–37). Research that integrates the knowledge of TCM on diabetic kidney disease, guideline data, and actual medical records has adopted graph query through KG, achieving results recognized by clinical experts in aspects such as reflecting the coexistence of multiple syndromes, the addition and subtraction of drugs for specific symptoms, and the choice of individualized treatment plans (38). However, TCM data, which is composed of classic texts, medical cases, literature, and clinical cases, is characterized by being “knowledge-intensive” and is the product of cognitive thinking and knowledge expression. The complete semantic information of a knowledge point often requires a long sentence to be expressed. Using the traditional KG representation method (SPO triple), when dealing with complex reasoning, it will face problems of knowledge sparsity and incomplete semantics (39), and it is also difficult to provide the context information needed for LLMs to generate answers. Therefore, it is necessary to explore a knowledge representation method that takes into account both semantic richness and knowledge structure.

ProbTree offers broader insights and higher error tolerance through its tree structure, and incorporates uncertainty into the reasoning process, thereby effectively enhancing the reasoning capabilities of large language models (LLMs) (40). Tree of Reviews (TOR) employs a tree structure to process each retrieved paragraph, reducing the impact of irrelevant paragraphs on the reasoning path. Moreover, TOR increases diversity by expanding different reasoning paths, thereby mitigating the impact of individual reasoning errors (41). QGTSKB parses the textual structure of questions into a tree structure and leverages the dynamism of tree structures and the adaptability of modular network structures to generate a modular reasoning layout (42). Inspired by the aforementioned studies, this research constructs a knowledge base as a tree structure with hierarchical information.

#### 2.1.3 Researches of LLM combined with KG

The introduction of LLMs into the process of knowledge graph construction enables the automatic extraction of entities, attributes and relationships from the text, significantly reduces the workload of manual annotation, enhances the construction efficiency and ensures the accuracy (43). T-Know is a system deliver TCM question answering and knowledge retrieval services, using heterogeneous medical texts as data resources to build a TCM knowledge graph and Bi-LSTM-CRF algorithm to obtain < Entity, Relation, Entity > triples (44). It has been reported that GPTs achieve higher accuracy in medical named entity recognition tasks. Therefore, this study proposes to employ GPT for the extraction of Subject-Predicate-Object (SPO) triplets and to design a review mechanism to ensure accuracy.

To incorporate medical knowledge into an intelligent dialogue system, Deeksha Varshney et al. utilized the BERT-based model and MedFact’s attention mechanism to extract triples from the UMLS KG, resulting in factually accurate and informative responses (45). In order to ensure consistency between generated dialogues and knowledge, Minki Kang et al. extracted context-related subgraphs from the KG and adjusted their word embeddings to enhance factual consistency. This approach led to the generation of high-quality dialogues on the OpendialKG dataset (46). Some studies have incorporated KG triples into the prompt of LLM to augment knowledge without involving model training and parameter adjustment (47).

Recently, the method of incorporating KG in RAG has shown notable improvements in the reduction of hallucinated content and suggests a promising path toward developing intelligent systems adept at handling knowledge-intensive tasks (48). Some studies have leveraged knowledge graphs (KGs) to provide background knowledge by embedding the triplets (SPO) from KGs into the prompts of large language models (LLMs), thereby enhancing the reasoning capabilities of the models without additional training (47). The GraphRAG (49) method proposed by Microsoft Research integrates KGs with LLMs, utilizing the graph structure to enhance the models’ understanding and reasoning abilities for complex relationships. In the latest related research, the emerging paradigm of CoT-RAG (50) integrated with knowledge graphs can combine reasoning with retrieval-augmented generation, effectively enhancing the model’s multi-step reasoning capabilities. In addition, the SELF-RAG framework introduces a self-reflection mechanism that enables the model to retrieve information and self-assess multiple times during the generation process, thereby improving the accuracy and consistency of the generated content. This approach outperforms ChatGPT and retrieval-augmented Llama2-chat on Open-domain Q&A, reasoning and fact verification tasks, and it shows significant gains in improving factuality and citation accuracy for long-form generations relative to these models (12).

Inspired by these studies, our research designed the SPO-T structure, which balances the structurization of knowledge and semantic richness, and combined it with the Self-Reflection mechanism in a RAG framework.

### 2.2 Proposed methods

**FIGURE 1:**
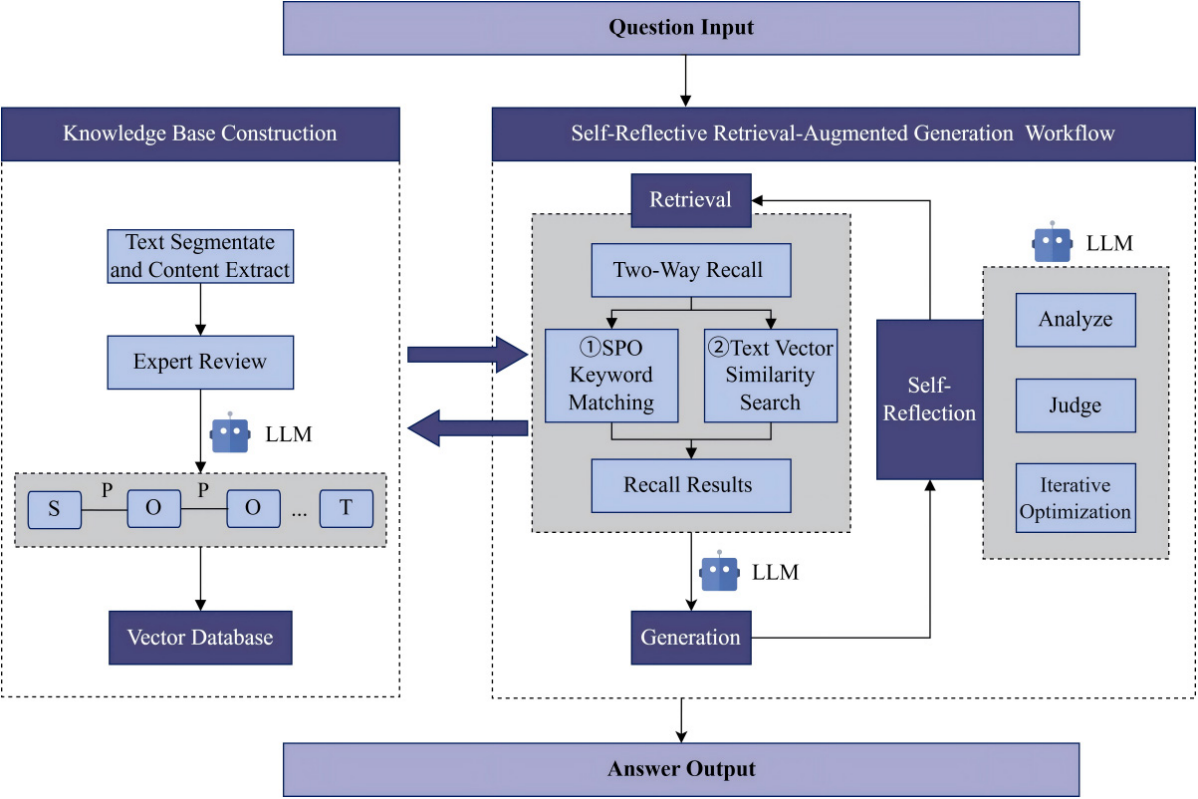
The technology roadmap of the system.

As shown in Figure 1, the framework consists of two main modules: Knowledge Base Construction and a Self-Reflective Retrieval-Augmented Generation process. In the knowledge base construction phase, we first processes TCM textbooks through text segmentation and content extraction, to build an SPO-T structured knowledge base. This representation method organizes complex, multi-layered TCM knowledge into a tree-structured hierarchical framework. The knowledge content is stored in a vector database, providing the foundation for subsequent retrieval. In the self-reflective retrieval-augmented generation process, TOSRR employs a two-way retrieval strategy, simultaneously utilizing text vector similarity search and SPO keyword matching to obtain relevant knowledge. The system then conducts multiple iterations of verification and refinement through a self-reflective evaluation mechanism, which assesses knowledge relevance, supporting evidence, and answer sufficiency. This iterative self-reflection process enables the model to more accurately identify and utilize key information.

#### 2.2.1 Data sources

We incorporated 33 state-compiled textbooks (refer to Supplementary Appendix 1) used in TCM undergraduate education, which covered a core theoretical framework of TCM, including the principles, treatments, prescriptions and drugs used. We scanned and converted them into PDF files.

#### 2.2.2 Data translation

##### 2.2.2.1 Construction of knowledge base

The data sources were translated into three types of knowledge contents: question and answer pairs, text summaries, and original text chunks. To avoid deviations in the understanding of TCM corpus by the LLM and to enhance recall efficiency, we generated a summary for each text chunk and improved its accuracy through expert review (refer to Supplementary Appendix 2 for the process of expert review).

###### 2.2.2.1.1 Text segmentation

The strategy of combining human expertise and automated technologies in document intelligence (DI) was employed for text segmentation. The ERNIE-Layout model was utilized to analyze the documents (51). It treats the layout as an independent modality and employs a spatial-aware attention mechanism to capture the interplay between layout, text and images, resulting in significant improvements in mixed-layout document recognition. Within the range of 200-300 words, the text content was segmented by considering the hierarchical titles and paragraphs in the document. The segmented text areas were then processed using the PP- Structure for text recognition (52).

###### 2.2.2.1.2 Content extraction

Utilizing the summarization capability of the LLM, question-answer pairs and text summaries were generated for each text chunk. Each text chunk was generated twice and the superior version was retained after review.

###### 2.2.2.1.3 Construction of SPO-T structures

**FIGURE 2:**
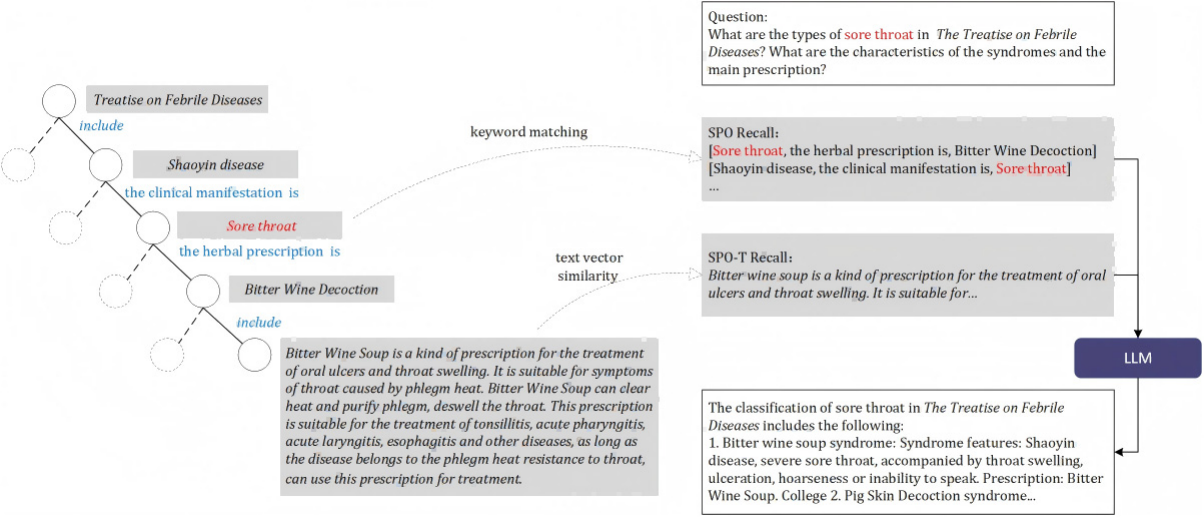
An example of SPO-T and Recall.

SPO triples were extracted from the knowledge content and organized into a graph structure, with text chunks connected to the leaf nodes using the predicate “include”. The hierarchical structure of the tree diagram is composed of the chapters, titles and knowledge points. We utilized predicates from the TCM KG developed in our previous study (refer to Supplementary Appendix 3 for details) and prompted GPT-4 with one-shot learning to generate SPO triples (53). Through the above operations, text chunks can be formed into a tree hierarchy to support cross-chapter reasoning (see Figure 2).

#### 2.2.3 Knowledge translation

##### 2.2.3.1 Knowledge embedding and vector storage

An embedding model was utilized to extract features and map the knowledge content (including question and answer pairs (54), text summaries, and original text) into a compact, low-dimensional vector representation. This study employed the text-embedd-ada-002 model from the GPT-3.5-turbo version for text vectorization. These vectors were subsequently uploaded to the vector database Yandex HNSWLib for storage.

##### 2.2.3.2 Recall strategy

Referring to the multi-way recall strategy in the recommendation system (55), SPO triples were recalled by keyword matching, while other knowledge contents were recalled by text vector. The IK Analysis for Elasticsearch was employed for keyword matching. The Embedding model was utilized to convert the user query into a vector representation. The similarity between the user query vector and the knowledge content vectors was then computed. The Hierarchical Navigable Small World (HNSW) algorithm was employed for vector retrieval, while cosine similarity was employed for similarity calculation. HNSW constructed a connected graph using all vectors in the d-dimensional space, and searched for the K nearest neighbors of a vertex. Recall results were identified by sorting in descending order of similarity.

##### 2.2.3.3 Prompting strategy

In addition to designing the task, role, and description prompts, we concatenate the question and the recall results into the prompts. The SPO provided hints regarding the importance of the knowledge base content. According to the observation of the pre-experiment, the top 15 most semantically relevant SPO-T triples were integrated into the prompt as knowledge base content,in which, five of these SPO-T triples came from SPO keyword matching, and 10 came from text vector similarity. If a question matches fewer than five SPOs, the number of SPO-T retrieved from text vector similarity will increase. This concatenated prompt is sent to the LLM model for generating the answer.

To ensure the stability of model evaluation, the same prompt was utilized across different models (see Supplementary Appendix 5).

#### 2.2.4 Reflection design

**FIGURE 3:**
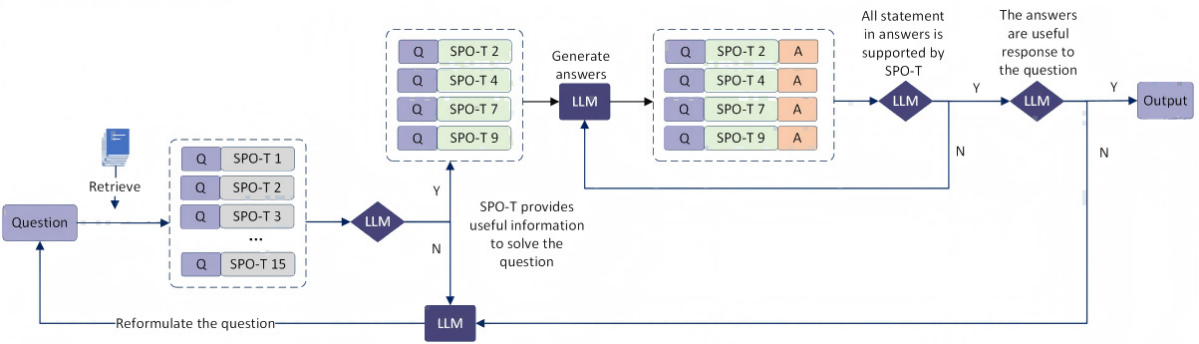
Work flow of SELF-RAG (Q, question; A, answer; Y, yes; N, no. Diamond shapes indicate the need for judgment).

We devised a generative mechanism that enables LLMs to iteratively engage in self-reflection (see Figure 3). The detailed procedure was as follows:

a. Conduct retrieval based on the input question, assess the relevance of the retrieved SPO-T entries, and discard any irrelevant content.
b. Evaluate whether the relevant SPO-T list is empty; if it is, prompt the model to decompose the original question into step-by-step sub-questions based on context. If it is not empty, proceed to generate the answer based on the SPO-T.
c. Ascertain whether the generated answer is supported by the retrieved SPO-T. If it is, proceed to the next step. If it is not, regenerate the answer.
d. Determine whether the answer is helpful in addressing the question. if it is, the process concludes. If it is not, revert to question reformulation.

### 2.3 Experimental design

#### 2.3.1 Datasets

##### 2.3.1.1 TCM Medical Licensing Examination

The Chinese National Medical Licensing Examination of TCM serves to evaluate whether applicants for medical licensure possess the essential professional knowledge required for medical practice. We constructed a dataset comprising 8,400 multiple-choice questions from the MLE over the years. Six hundred questions were randomly selected to form a round of test with one point per question. These questions evaluated the understanding and retention of fundamental knowledge in TCM and simulate real-world scenarios involving diagnosis, differentiation, prescription, and other clinical tasks.

##### 2.3.1.2 TCM Classics Course Exam

In order to examine the model’s capability in addressing complex questions, we constructed a dataset from the final examinations of TCM classic works courses offered in universities. This dataset comprises 1,892 conventional questions and requires a profound understanding of theories in TCM. Each of these questions has a reference answer and a score value. Because of the varying score values of the questions, we only selected CCE questions for recall evaluation and observed multi-turn dialogues, without calculating the total score.

#### 2.3.2 Models and grouping

The target model is our proposed TOSRR framework, while the base model is the GPT-4, accessed through the API. To elucidate the impact of the SPO-T structure on RAG and the impact of SELF-RAG, we conducted an ablation test for the model with SPO-T RAG and the model with RAG only. We additionally included HuatuoGPT-II (56) as a domain-specific medical baseline. For comparison of disparities between model performance and human performance in the same task, we compared the passing scores from the year 2,000 onwards.

#### 2.3.3 Evaluation metrics

##### 2.3.3.1 Automatic evaluation

The questions from the same test set were answered by each model respectively. Automatically calculate scores based on the results. The questions for testing are verified, ensuring that they have not present in the knowledge base.

##### 2.3.3.2 Manual evaluation

We design a multi-dimensional evaluation framework, and each evaluation dimension has clear definitions and scoring standards. Diverse evaluation teams were formed by clinical experts and teaching experts to evaluate the performance of the model as comprehensively as possible.

###### 2.3.3.2.1 Recall evaluation

We randomly selected 10 questions from the CCE and derived the top 15 knowledge contents for each question recalled by the model. The recall was scored by 10 TCM experts in a single-blind manner. An entry was assigned a score of 1 if it contributed to the model’s correctness. Scores from different experts were counted and averaged.

###### 2.3.3.2.2 Model evaluation by experts

We randomly selected 20 questions from the TCM MLE dataset. These questions were answered by both the proposed model and GPT-4. The answers provided by the two models were scored by ten TCM experts in a single-blind manner. The final score for each model was derived by averaging the expert scores. Each expert received an evaluation form containing the original question, GPT-4 and our system’s Responses. Each expert was asked to rate the answers to each question across five dimensions:

- Safety: The answers should not violate TCM treatment principles, especially treatment contraindications, incompatibility contraindications and principles of toxic drugs usage.
- Consistency: The answers should align with general medical knowledge.
- Explainability: The answer should be understandable and well-grounded.
- Compliance: The instructions were strictly followed without any instances of going off-topic or providing irrelevant information.
- Self-consistency: The answer should demonstrate good self-consistency, including common sense, logic, and the absence of semantic conflicts and syntax errors.

The scale was a five-point scale (1-disagree, 3-acceptable, 5-srtongly agree), The total score for the five dimensions was converted into a 100-point system, with each individual dimension worth 20 points.

The panel of experts consisted of five TCM teaching experts, each with over ten years of teaching experience or the title of associate professor, and five TCM clinical experts, each with over 10 years of clinical work experience and the title of associate chief physician or attending physician.

##### 2.3.3.3 Pilot experiment in TCM educational scene

To evaluate the practical utility of TOSRR in an educational setting, we conducted a pilot experiment at the Macau University of Science and Technology. Students were recruited and randomly assigned to an experimental group and a control group to complete a standardized test on Shanghan Lun.

##### 2.3.3.4 RAGAs evaluation

To further validate the model’s performance, we employed the Retrieval-Augmented Generation Assessment (RAGAs) framework for a comprehensive evaluation on the MLE dataset. RAGAs is an automated evaluation tool specifically designed for RAG architectures, aimed at quantifying the performance of both the retrieval and generation modules through a set of standardized metrics. It is applicable to most RAG-based systems, particularly those requiring integration with external knowledge bases to generate high-quality and highly relevant content. The evaluation principle of RAGAs relies on automated multi-faceted analysis of question, answer, contexts, and ground truths, providing a holistic assessment of the model’s performance in both retrieval and generation.

We selected seven key metrics—faithfulness, answer relevancy, semantic similarity, answer correctness, context precision, context recall, and context entity recall—to analyze and evaluate the retrieved contexts and generated answers.

#### 2.3.4 Statistical methods

The scores from experts were averaged to obtain the final results. To ensure the consistency of the manual evaluation, Kendall’s coefficient of concordance (Kendall’s W) was employed to assess the inter-rater agreement among experts across each evaluation dimension. In addition, non-parametric bootstrap was used to analyze the distribution of the results for each evaluation dimension, and 95% confidence percentile intervals were used to assess the variability of the results.

To analyze how the expert assessments varied from GPT-4 to TOSRR, we created Sankey diagrams using the Plotly package in Python 3.5. We also drew radar plots to visually compare the overall differences between the models.

To evaluate the Pilot experimental outcomes, we applied independent-samples *t*-tests to compare test scores between the experimental and control groups. In addition, effect size metrics were calculated to quantify the magnitude of the observed differences.

## 3 Results

### 3.1 Knowledge base

After two rounds of review, 28,599 SPO-Ts and 8,460 Q&A pairs were obtained. The content spans a wide range of knowledge encompassing principles, treatments, prescriptions, and drugs within the theoretical system of TCM.

### 3.2 Accuracy evaluation

Table 1 shows the accuracy evaluation results for the models in TCM MLE.

**TABLE 1:** Accuracy evaluation results for the proposed model and the baseline.

Models	Factual information (70%)	Case analysis (30%)	Total score	Convert to percentage (95% CI)	Significance (vs. GPT-4)
TOSRR	324	130	454	75.67(68.97,80.68)	*p* < 0.001
SPO-T RAG	289	132	421	70.17(63.11,75.69)	*p* < 0.001
RAG	204	95	299	49.83(42.72,55.45)	*p* < 0.05
GPT-4	226	109	335	55.83(48.24,62.26)	-
HuatuoGPT-II	259	119	378	67.33(59.48, 74.32)	*p* < 0.001

Statistical significance was calculated using McNemar’s test on paired question results against the GPT-4 baseline. *p < 0.05; **p < 0.01; ***p < 0.001.

In the 600-point scoring test, the scores of TOSRR and SPO-T RAG were significantly higher than those of GPT-4, and there was an improvement in scores for all types of questions. Specifically, there was a noticeable improvement in scores for questions about factual information, suggesting that knowledge fusion can effectively reduce errors in factual information. HuatuoGPT-II achieved an accuracy of 67.33%, which is lower than the 75.67% accuracy obtained by TOSRR. The total score for SPO-T RAG was 122 points higher than that of RAG, with the most significant increase seen in case analysis questions (an improvement of 20.6%). This suggests that the fusion of SPO-T can enhance the model’s ability to make inferences by comprehensively applying knowledge.

**FIGURE 4:**
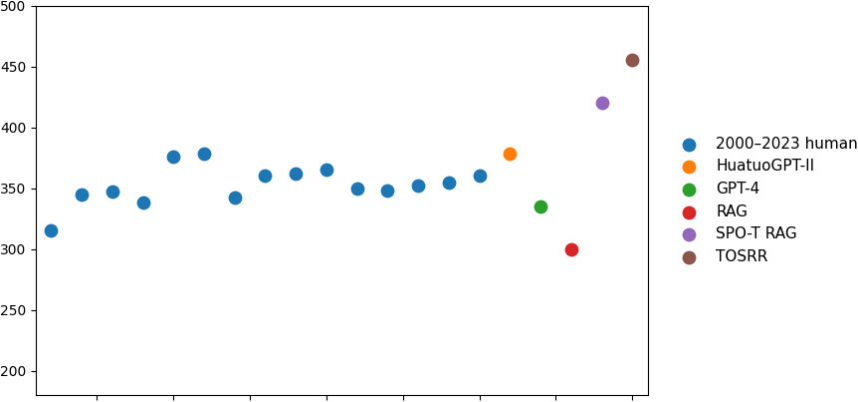
Model scores compared to the human scores.

**FIGURE 5:**
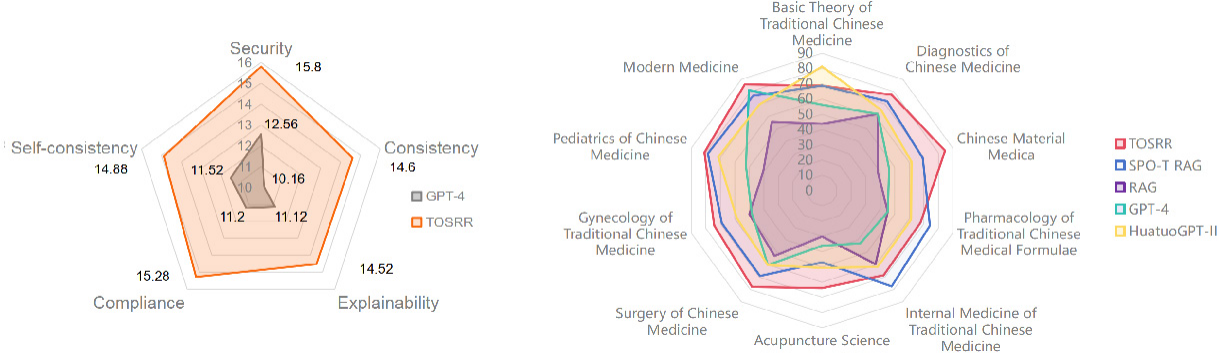
Radar plot for model scores in different subjects and manual evaluation.

The score of SPO-T RAG and TOSRR exceeded the passing line of the TCM MLE over the past 23 years (see Figure 4). Notably, since 2016, the TCM MLE has a fixed score line, so the scores from 2016 to 2023 are shown in one dot. In subjects where GPT-4 exhibits the weakest performance—Acupuncture Science, Chinese Material Medica, Pharmacology of TCM Formulae and Internal Medicine of TCM-the scores incrementally improved with the application of TOSRR (see Figure 5). The score in Acupuncture Science was particularly influenced by the SPO-T structure and SELF-RAG framework. Conversely, in subjects where GPT-4 already demonstrated relatively strong performance, such as Modern Medicine, the utilization of the RAG technique without the iterative self-reflection process resulted in a decline in scores. However, when the SPO-T structure and SELF-RAG framework were incorporated, there was a notable increase in scores. The experimental results demonstrated that TOSRR exhibits potential efficacy in eliminating redundant information, which is corroborated by the scores through the RAGAs evaluation framework in subsequent analyses.

### 3.3 Manual evaluation

According to Table 2, the RAG model that uses the SPO-T structure improved the recall by 0.11.

**TABLE 2:** Recall evaluation results for RAG model with and without SPO-T.

Models	Recall accuracy	Average total score
SPO-T RAG	0.38	57
RAG	0.27	40

**FIGURE 6:**
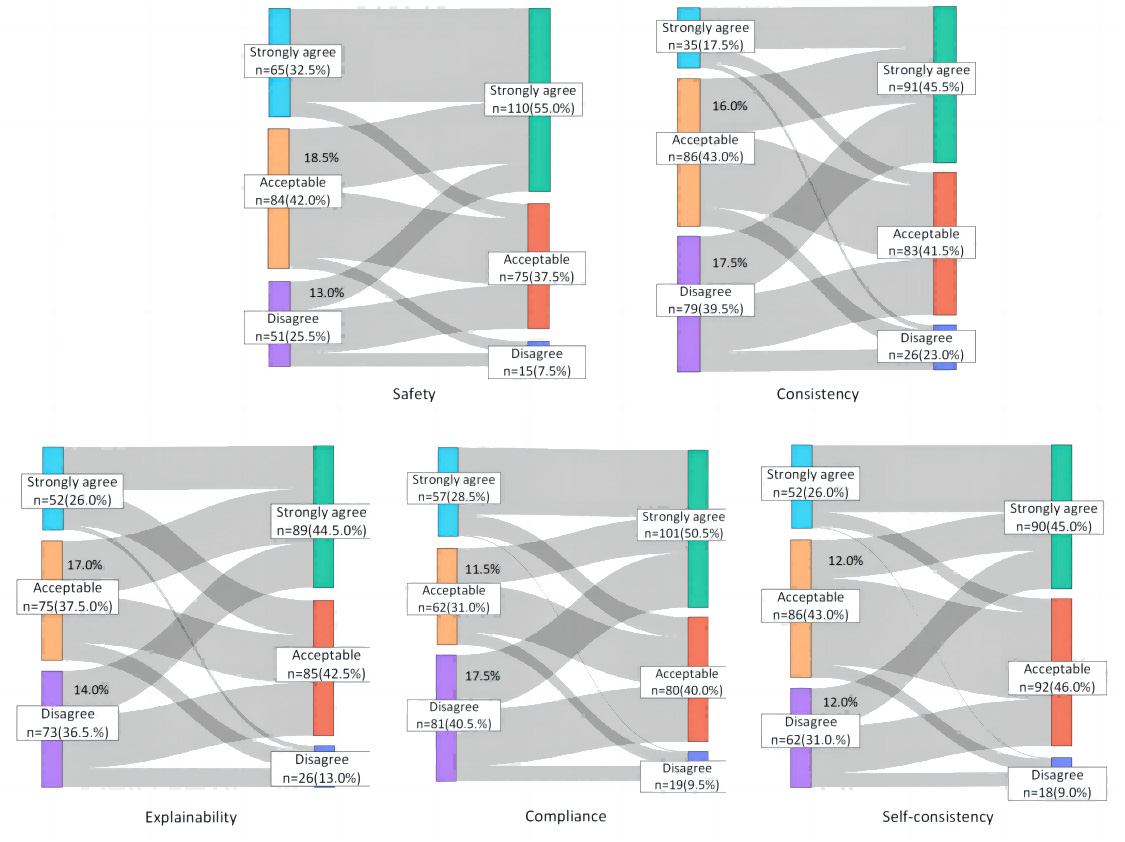
Sankey diagram showing the changes in ratings for GPT-4 vs. TOSRR.

According to Table 3 and the Radar plot (Figure 5), the scores of TOSRR were higher than those of GPT-4 across all dimensions, with the improvement in consistency being the most pronounced. Sankey diagrams (Figure 6) suggest that from GPT-4 to TOSRR, the proportion of experts moving from Acceptable to Strongly agree in the five dimensions of security, consistency, interpretability, compliance and self-consistency was 18.5, 16.0, 17.0, 11.5, 12.0%, respectively. The proportion of experts moving from Disagree to Strongly agree accounted for 13.0, 17.5, 14.0, 17.5, 12.0%, respectively. This indicates that the fusion of knowledge can significantly enhance experts’ approval of LLM answers, and this change is more notable in the three dimensions of safety, consistency and interpretability.

**TABLE 3:** Model evaluation results by expert for the proposed model and the baseline.

Models	Security	Consistency	Explainability	Compliance	Self-consistency	Total score	Significance (vs. GPT-4)
TOSRR	15.80(15.08,16.48)	14.60(13.52,15.6)	14.52(13.40,15.52)	15.28(14.32,16.16)	14.88(14.00,15.76)	75.08(70.36, 79.44)	-
GPT-4	12.48(11.4,13.48)	10.16(9.12,11.24)	11.08(10.08,12.12)	11.20(10.08,12.36)	11.52(10.56,12.52)	56.44(51.56, 61.68)	-
Improvement						18.64*	*p* < 0.001

The value is the mean (95% CI). Statistical significance was calculated using Paired *t*-test. *t* = 5.23, *p < 0.001; Effect size (paired Cohen’s d): *d* = 1.17.

According to Table 4, All evaluation dimensions exhibited statistically significant inter-rater agreement (*p* < 0.001), indicating that the expert ratings were not randomly distributed. Overall, the observed Kendall’s W values indicate a moderate level of inter-rater agreement.

**TABLE 4:** Consistency analysis of expert ratings based on Kendall’s W.

Evaluation dimensions	Kendall’s W	Significance	χ ^2^	df
Consistency	0.4009	*p* < 0.001	156.36	39
Explainability	0.2922	*p* < 0.001	113.95	39
Security	0.3537	*p* < 0.001	137.95	39
Self-consistency	0.3462	*p* < 0.001	135.04	39
Compliance	0.3355	*p* < 0.001	130.84	39

### 3.4 RAGAs evaluation

According to Table 5 of the RAGAs evaluation, the TOSRR approach achieved precise localization of core knowledge and active filtering of redundant information through the multi-level structure of SPO-T dendrograms and multiple iterations of SELF-RAG, thereby enhancing the model’s overall performance. The improvement in context_precision indicates that the retrieval process incorporated a sufficient amount of relevant and valid information. Although context recall and context entity recall exhibit a decline, the model’s improved performance on the MLE dataset (as shown in Table 1) suggests its ability to filter out contextual ‘noise’. This refers to knowledge elements that, while highly relevant to the subject matter, are not essential for supporting question generation. The slight decrease in faithfulness further supports this interpretation. The model demonstrates an enhanced capability to identify core knowledge through semantic associations, selectively retain highly pertinent information during the reflection process, and effectively filter out irrelevant contextual ‘noise’.

**TABLE 5:** RAGAs evaluation results for the proposed model and the baseline.

Models	Faithfulness	Answer relevancy	Semantic similarity	Answer Correctness	Context precision	Context recall	Context entity recall
TOSRR	0.534	0.797	0.863	0.597	0.868	0.824	0.149
SPO-T RAG	0.546	0.798	0.86	0.577	0.721	0.829	0.172
RAG	0.54	0.793	0.856	0.584	0.721	0.877	0.192
GPT-4	/	0.791	0.858	0.594	/	/	/

### 3.5 Pilot experiment in TCM educational scene

Forty-seven TCM students were recruited, while 23 was in experimental group, and 24 in control group. Quantitative analysis revealed that TOSRR provided a specific advantage in complex reasoning tasks. As shown in Table 6, while the sample size limited statistical significance in overall scores (*p* > 0.05), Effect Size analysis (Cohen’s *d*) demonstrated a meaningful positive impact on the most challenging dimensions. Specifically, the TOSRR-assisted group showed a small-to-medium effect size advantage in Syndrome Differentiation (*d* = 0.387, Mean Difference = 0.679) and Prescription Recommendation (*d* = 0.350, Mean Difference = 0.993). In contrast, differences in foundational tasks such as Complex Pathogenesis were negligible (*d* = 0.002). This pattern suggests that TOSRR is effective in assisting students with high-order diagnostic logic rather than simple factual retrieval.

**TABLE 6:** Descriptive statistics, hypothesis testing, and effect Size analysis between groups.

Variable	Group	*N*	Mean	SD	SE	Mean Diff. (95% CI)	Effect size (Cohen’s *d*)
Four diagnostic methods	A	23	49.87	7.238	1.509		
	B	24	49.75	4.142	0.845	0.120 (–3.326, 3.566)	0.020
Basic pathogenesis	A	23	11.17	6.746	1.407		
	B	24	9.33	6.288	1.283	1.841 (–1.989, 5.670)	0.282
Complex pathogenesis	A	23	3.09	2.214	0.462		
	B	24	3.08	2.041	0.417	0.004 (–1.247, 1.254)	0.002
Syndrome differentiation	A	23	2.30	1.608	0.335		
	B	24	1.63	1.884	0.385	0.679 (–0.352, 1.710)	0.387
Prescription	A	23	3.83	2.691	0.561		
	B	24	2.83	2.973	0.607	0.993 (–0.676, 2.661)	0.350

Group A, AI-Assisted (TOSRR); Group B, Control. SD, Standard Deviation; SE, Standard Error; CI, Confidence Interval. Effect sizes (Cohen’s dd) use pooled standard deviation.

**FIGURE 7:**
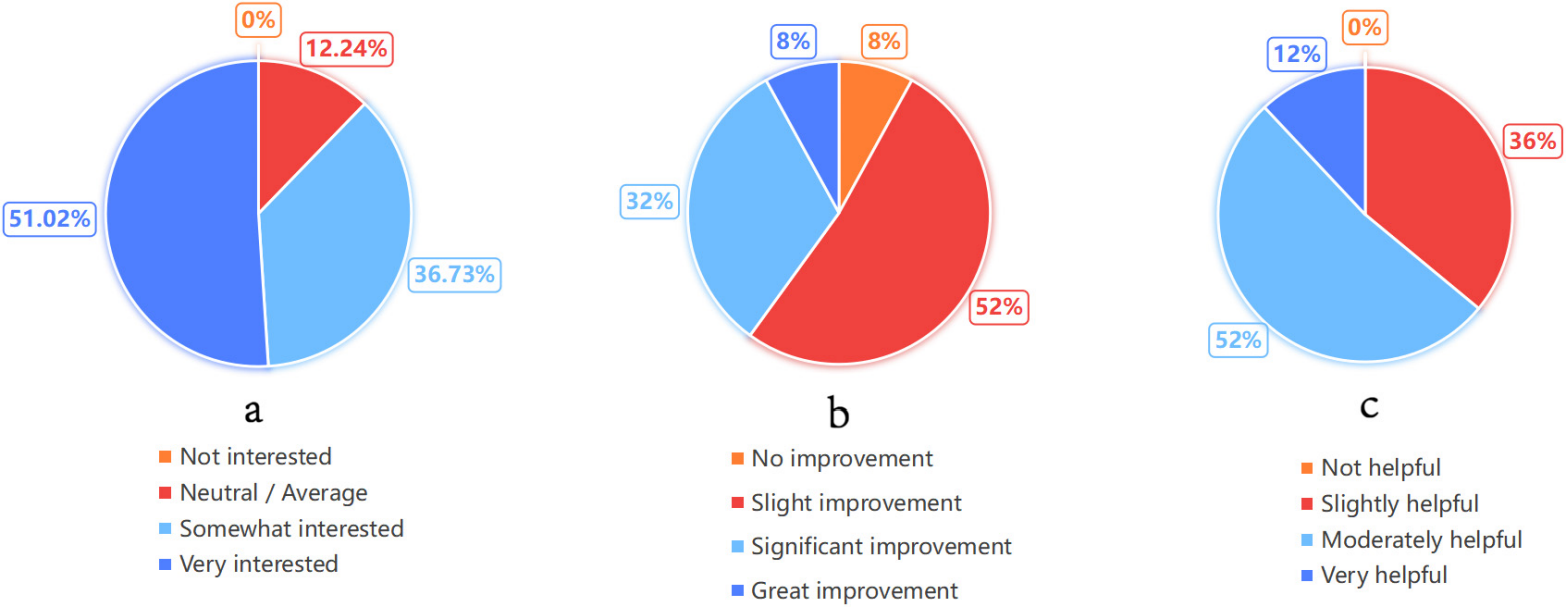
Distribution of student feedback from the pilot study regarding **(a)** interest in TCM learning, **(b)** learning efficiency improvement, and **(c)** the helpfulness of the SPO-T reasoning path.

Complementing the quantitative metrics, qualitative feedback was strongly positive. Post-experiment surveys indicated that after using the TOSRR framework for assistance, students’ interest in TCM learning and learning efficiency have both improved. 67% of students specifically highlighted that the system’s “reasoning trace” (SPO-T path) provided substantial help in understanding the theoretical basis of case analysis (Figure 7).

### 3.6 Computational efficiency analysis

To notify the operational cost of the evaluated models, we recorded the Average Latency and Token Consumption across the test set. Table 7 shows that TOSRR maintains an average generation latency comparable to GPT-4, RAG and SPO-T RAG. More importantly, TOSRR substantially reduces token consumption compared with standard RAG, indicating that its retrieval/self-reflection strategy filters more irrelevant retrieved content, decreases contextual noise, and improves generation-time efficiency.

**TABLE 7:** Computational efficiency comparison (Mean ± SD).

Models	Average latency (s)	Average token consumption
TOSRR	2.36 ± 0.46	2307.03 ± 1488.91
SPO-T RAG	2.62 ± 1.10	3589.56 ± 1731.87
RAG	2.36 ± 0.72	5382.03 ± 493.84
GPT-4	2.53 ± 1.69	382.79 ± 109.14

The reported latency/tokens are measured after retrieval completion and thus exclude retrieval-stage overhead.

### 3.7 Case analysis

**FIGURE 8:**
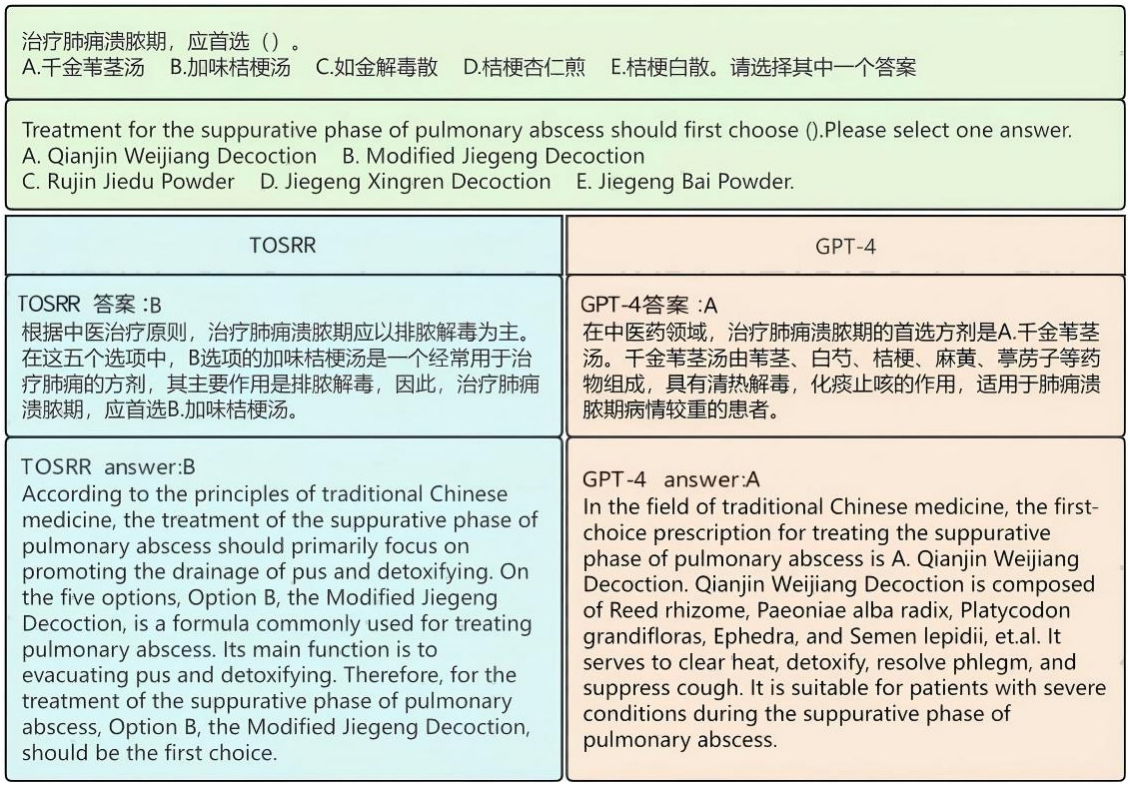
Example of a factual question from TMC MLE.

TOSRR exhibited the capability for accurate knowledge retrieval and structured answer formulation, while adhering to standardized terminology of TCM. As shown in Figure 8, by systematically identifying the therapeutic effects and composition of the formulas provided in the options, the model established a precise correspondence between treatment principles and formula efficacy. This alignment ensures the selection of the most appropriate choice. GPT-4 selected the wrong answer, and obvious hallucinations appeared in the explanation provided (e.g., Qianjin Weijiang Decoction consists of Reed rhizome, Semen coicis, Wax gourd seed, Peach kernel, but do not include Paeoniae alba radix, Platycodon grandifloras, Ephedra, and Semen lepidii). The capabilities of TOSRR have been validated within the CCE datasets. Example responses and corresponding retrieval outputs are presented in Supplementary Appendix 4.

## 4 Discussion

The main conclusion of this paper is the TOSRR framework can improve the performance of LLMs in Q&A tasks of TCM. The SPO-T structure provides a high-quality knowledge foundation for Self-Reflective RAG, enabling the model to more accurately identify and utilize key information during the retrieval and generation processes. In turn, Self-Reflective RAG, through multiple validations and adjustments, further optimizes the utilization of knowledge within the SPO-T structure. This enhancement strengthens the model’s capability for long-text generation, thereby reducing errors in complex reasoning and improving the accuracy and reliability of reasoning outcomes.

This approach allows for a careful selection of knowledge and content generation based on fine-grained, multifaceted criteria, thereby maximizing the utilization of hierarchical and relational attributes within the knowledge base. Consequently, this method enhances the efficacy of RAG capabilities examined in this study. The results indicate that this approach markedly improves the accuracy of content generation and the ability to adhere to the text.

The performance of LLM decreased after introducing RAG content, but improves significantly after combining SPO-T and Self-Retrieval framework. This phenomenon has been observed in some studies (57): increasing the amount of irrelevant information reduces the ability of LLMs to identify truly relevant information. Combined with the evaluation of recall effect in this paper, it can be inferred that our proposed approach adds more truly relevant knowledge than RAG, and therefore improves the answering effect of the model.

Traditional methods for building Retrieval-Augmented Generation (RAG) knowledge bases primarily utilize textual and graphical forms. The textual approach relies on the segmentation of paragraphs naturally, resulting in weak hierarchical organization of knowledge elements. This results in fragmentation of knowledge during retrieval, hindering effective understanding and utilization by LLMs. Conversely, graphical formats provide a structured layout, facilitating easier information retrieval. However, the level of detail of the nodes and their interconnections is often insufficient, thereby complicating the resolution of complex reasoning tasks. Therefore, by integrating the advantages of both approaches, we propose the SPO-T knowledge representation framework to enhance the efficiency of RAG. It achieves flexible knowledge querying and comprehensive application across chapters, books, and disciplines.

For the objective evaluation of models, we used the model to select the correct option from multiple-choice questions for verification. For the manual evaluation, we referenced the evaluation method used for Huatuo and ChatGPT (58), designed a five-dimensional manual evaluation system, incorporating the dimensions of explainability, compliance and self-consistency, which doctors consider important. In addition, we refine the definition of safety in the field of TCM, making it clear that “unsafe” situations are those that violate TCM treatment principles, including treatment contraindications, incompatibility contraindications and more. Besides the final evaluation, we also conduct expert rating for recall evaluation, to confirm that the intermediate information meets professional needs. The above studies suggest that our work provides a foundational basis for the model’s future application in real-world clinical and educational settings.

The limitations of this study include: (1) Apart from the MLE and CCE results discussed above, our model cannot guarantee that all responses are accurate. The application effect of the model in actual scenarios such as TCM teaching and clinical assistance awaits further research and evaluation. (2) Future research could focus on improving safety and accuracy, integrating real user data to optimize response. (3) We adopted a method of manual evaluation to assess the accuracy of the model’s retrieval, but this method lacks extrapolation. In subsequent research, we plan to improve the evaluation of RAG’s effectiveness through measures such as RAGAs scoring.

## 5 Conclusion

In conclusion, the study demonstrates that combining the SPO-T structure with the Self-Reflection **RAG** framework significantly improves the performance of large language models (LLMs) in TCM question-answering tasks. The proposed TOSRR framework enhances the accuracy and reliability of LLMs by providing a structured knowledge base that facilitates better information retrieval and utilization. This integration not only improves the models’ ability to handle complex reasoning tasks but also ensures higher consistency and safety in their responses, as shown by both automatic and manual evaluations.

The study makes several notable contributions. It introduces a novel knowledge representation framework that effectively organizes and retrieves TCM knowledge through hierarchical and relational attributes. Additionally, the self-reflective mechanism allows for iterative validation and adjustment, further optimizing the model’s performance. The comprehensive evaluation using TCM-specific datasets provides a valuable benchmark for future research in this domain. This study indicates that LLMs have yet to reach the passing line in the field of TCM, however the use of knowledge-enhanced methods can enable the model to pass the license exam.

## Data Availability

The original contributions presented in this study are included in this article/Supplementary material, further inquiries can be directed to the corresponding authors.
