# Supplementary material for: Improving TCM question answering through tree-organized self-reflective retrieval with LLMs
Source: Front Med (Lausanne). 2026 Mar 12;13:1752778. doi: 10.3389/fmed.2026.1752778 (PMC13019696; doi:10.3389/fmed.2026.1752778)
Supplement: Supplementary file 1 [file Data_Sheet_1.docx]

Appendix 1: 33 state-compiled textbooks

| **Index** | **Data Source** | **Term Number** |
| --- | --- | --- |
| 1 | Infectious Diseases | 2867 |
| 2 | Pediatrics | 3725 |
| 3 | Pharmacology of Traditional Chinese Medical Formulae | 2480 |
| 4 | Obstetrics and Gynecology | 3552 |
| 5 | Synopsis of Golden Chamber | 928 |
| 6 | Selected Reading of Huangdi Neiing | 329 |
| 7 | Internal Medicine | 6874 |
| 8 | Selection of Treatise on Febrile Diseases | 679 |
| 9 | Neurology | 3443 |
| 10 | Science of Tuina | 1816 |
| 11 | Surgery | 5166 |
| 12 | Science of Seasonal Febrile Disease | 539 |
| 13 | Pediatric Tuina | 1224 |
| 14 | Pharmacology | 2569 |
| 15 | Medical Ethics | 757 |
| 16 | Medical Imaging | 2508 |
| 17 | Acupuncture Science | 1278 |
| 18 | Diagnostics | 2812 |
| 19 | Integrated Traditional Chinese and Western Medicine Internal Medicine | 5470 |
| 20 | Chinese Material Medica | 681 |
| 21 | Pediatrics of Chinese Medicine | 1538 |
| 22 | Traditional Chinese Medicine Otorhinolaryngology | 1023 |
| 23 | Gynecology of Traditional Chinese Medicine | 2023 |
| 24 | Orthopedics of Traditional Chinese Medicine | 2003 |
| 25 | Basic Theory of Traditional Chinese Medicine | 837 |
| 26 | Internal Medicine of Traditional Chinese Medicine | 1371 |
| 27 | Traditional Chinese Dietary Therapy | 3388 |
| 28 | Surgery of Chinese Medicine | 1465 |
| 29 | Chinese Medicine | 3703 |
| 30 | Ophthalmology of Traditional Chinese Medicine | 2157 |
| 31 | Science of Health Maintenance of Traditional Chinese Medicine | 1186 |
| 32 | Chinese Traditional Medicine Scientific Diet | 2110 |
| 33 | Diagnostics of Chinese Medicine | 787 |

**References:**

1. LJ Li. Infectious Diseases. Beijing: People's Health Publishing House, 2018.

2. DZ Mu. Pediatrics. Beijing: People's Health Publishing House, 2018.

3. J Li. Pharmacology of Traditional Chinese Medical Formulae. Beijing: China Traditional Chinese Medicine Press, 2021.

4. X Xie. Obstetrics and Gynecology. Beijing: People's Health Publishing House, 2018.

5. YS Fan. Synopsis of Golden Chamber. Beijing: China Traditional Chinese Medicine Press, 2021.

6. SQ Zhai. Synopsis of Golden Chamber. Beijing: China Traditional Chinese Medicine Press, 2021.

7. JB Ge. Internal Medicine. Beijing: People's Health Publishing House, 2018.

8. QG Wang. Selection of Treatise on Febrile Diseases. Beijing: China Traditional Chinese Medicine Press, 2021.

9. JP Jia. Neurology. Beijing: People's Health Publishing House, 2018.

10. M Fang. Science of Tuina. Beijing: China Traditional Chinese Medicine Press, 2021.

11. MC Wu. Surgery. Beijing: People's Health Publishing House, 2018.

12. XH Gu. Science of Seasonal Febrile Disease. Beijing: China Traditional Chinese Medicine Press, 2021.

13. MJ Liu. Pediatric Tuina. Beijing: China Traditional Chinese Medicine Press, 2021.

14. WJ Zang. Pharmacology. Beijing: People's Health Publishing House, 2018.

15. MX Wang. Medical Ethics. Beijing: People's Health Publishing House, 2018.

16. K Xu. Medical Imaging. Beijing: People's Health Publishing House, 2018.

17. FR Liang. Medical Imaging. Beijing: China Traditional Chinese Medicine Press, 2021.

18. XH Wan. Diagnostics. Beijing: People's Health Publishing House, 2018.

19. ZQ Chen. Integrated Traditional Chinese and Western Medicine Internal Medicine. Beijing: China Traditional Chinese Medicine Press, 2021.

20. GS Zhong. Chinese Material Medica. Beijing: China Traditional Chinese Medicine Press, 2021.

21. X Zhao. Pediatrics of Chinese Medicine. Beijing: China Traditional Chinese Medicine Press, 2021.

22. P Liu. Traditional Chinese Medicine Otorhinolaryngology. Beijing: China Traditional Chinese Medicine Press, 2021.

23. XL Feng. Gynecology of Traditional Chinese Medicine. Beijing: China Traditional Chinese Medicine Press, 2021.

24. GC Huang. Orthopedics of Traditional Chinese Medicine. Beijing: China Traditional Chinese Medicine Press, 2021.

25. HX Zheng. Basic Theory of Traditional Chinese Medicine. Beijing: China Traditional Chinese Medicine Press, 2021.

26. MH Wu. Internal Medicine of Traditional Chinese Medicine. Beijing: China Traditional Chinese Medicine Press, 2021.

27. HF Shi. Traditional Chinese Dietary Therapy. Beijing: China Traditional Chinese Medicine Press, 2021.

28. HF Chen. Surgery of Chinese Medicine. Beijing: China Traditional Chinese Medicine Press, 2021.

29. JS Chen. Chinese Medicine. Beijing: People's Health Publishing House, 2018.

30. QH Peng. Ophthalmology of Traditional Chinese Medicine. Beijing: China Traditional Chinese Medicine Press, 2021.

31. LG Ma. Science of Health Maintenance of Traditional Chinese Medicine. Beijing: China Traditional Chinese Medicine Press, 2021.

32. MZ Xie. Chinese Traditional Medicine Scientific Diet. Beijing: China Traditional Chinese Medicine Press, 2021.

33. CD Li. Diagnostics of Chinese Medicine. Beijing: China Traditional Chinese Medicine Press, 2021.
