# Supplementary material for: Improving TCM question answering through tree-organized self-reflective retrieval with LLMs
Source: Front Med (Lausanne). 2026 Mar 12;13:1752778. doi: 10.3389/fmed.2026.1752778 (PMC13019696; doi:10.3389/fmed.2026.1752778)
Supplement: Supplementary file 2 [file Data_Sheet_2.docx]

##### Appendix 2: Expert review

**Text segmentation**

Five TCM teaching experts reviewed the text chunks. The review criteria included a complete knowledge framework, comprehensive content, and logical integrity within the chunks. The texts that did not meet the review criteria were subsequently corrected.

**Content generation**

Due to the noise present in the generated content, all knowledge contents were reviewed by five experts specializing in TCM teaching. A special internal system was designed for the review process and a detailed review strategy was formulated. The initial screening involved identifying and deleting clearly incorrect entries, a task conducted by twenty medical students. Content that was unclear and recommended for deletion was submitted to experts for confirmation. Five experts conducted a second screening to revise the knowledge contents. Refer to Table 1 for the categories of noise.

Table 1 Review criteria for knowledge contents.

| Categories | Operation | Noise category | Example |
| --- | --- | --- | --- |
| First screen | Delete | Literary Nonsense | "What type of syndrome does the excessive cold Chest stagnation(Jiexiong) syndrome belong to? It belongs to the syndrome of excessive cold." |
|  |  | Additional information needed to answer this question | "What evidence and treatment do the article suggest? Syndrome treatment with Guizhi Er Yuebi Yi Decoction" |
|  |  | Meaningless content | "The patient's clinical presentation was:..." (extracted from medical records in text) |
| Double screen | Revise | Error of fact | "Shaoyin disease can be treated with Dachengqi Decoction" is a factual error and should be corrected to "Yangming disease can be treated with Dachengqi Decoction" |
|  |  | Excessive omission of meaning | "The Taiyang Disease is treated by the purging method" omits the process of disease transmission, and is added as "When the Taiyang Disease transmitted to Yangming Disease after the wrong treatment, it can be treated by the purging method." |
|  |  | Supplementary Context | "This article", "this license", "this party", etc., supplement the specific information referred to |
|  |  | Confusion of concepts | "The clinical manifestation of GeGen Decoction is..." ", confused the name of the prescription and the name of the prescription-syndrome (Fangzheng), revised to "the clinical manifestation of Gegen Decoction Syndrome is..." |
