# Supplementary material for: Improving TCM question answering through tree-organized self-reflective retrieval with LLMs
Source: Front Med (Lausanne). 2026 Mar 12;13:1752778. doi: 10.3389/fmed.2026.1752778 (PMC13019696; doi:10.3389/fmed.2026.1752778)
Supplement: Supplementary file 3 [file Data_Sheet_3.doc]

Appendix 3: Candidate predicates from KG.

| 治疗原则是 | The treatment principle(s) is/are |
| --- | --- |
| 治疗措施是 | The treatment measure(s) is/are |
| 治疗部位是 | The treatment location(s) on the body is/are |
| 治疗 | Treat |
| 欲解时是 | The time when the disease starts to heal |
| 预后是 | The prognosis(ses) is/are |
| 特性是 | The characteristic(s) is/are |
| 临床表现是 | The clinical manifestation(s) is/are |
| 禁忌症是 | The contraindication(s) is/are |
| 禁忌是 | Prohibit |
| 鉴别诊断是 | Differential diagnosis(ses) is/are |
| 功效是 | The effect(s) is/are |
| 服药反应是 | Medication response(s) is/are |
| 方剂是 | The herbal prescription(s) is/are |
| 对应病机 | Corresponding pathogenesis |
| 导致 | Induce |
| 病因是 | The cause(s) is/are |
| 病位是 | The disease location(s) is/are |
| 病传是 | pathological transmission(s) |
| 包含 | Include |
