# Supplementary material for: Improving TCM question answering through tree-organized self-reflective retrieval with LLMs
Source: Front Med (Lausanne). 2026 Mar 12;13:1752778. doi: 10.3389/fmed.2026.1752778 (PMC13019696; doi:10.3389/fmed.2026.1752778)
Supplement: Supplementary file 4 [file Data_Sheet_4.doc]

**Appendix 4. Example of answer for TCM CCE datasets**

问题：请解释广义痰饮治疗原则：”温药和之”的含义（5分）

Question 1: Please explain the treatment principle of phlegm and retained fluid diseases: ‘harmonizing with the warm medicine’.

参考答案：温药能振奋阳气，开发腠理，通行水道；’和之’有两层含义：一是不可太过温燥，二是勿专于温补。即使用温药时，应视病情恰当配合行、消、开、导、清之品。

Reference answer: Warm medicine can motivate Yang qi, induce perspiration, diuresis; *Harmonizing* has two meanings: one is not too warm, and the other is not dedicated to warming. That is, when using warm medicine, it should be appropriate to cooperate with the medicine of promoting circulation, dispersing, diaphoresis, purgation and clearing according to the condition.

TOSRR答案：’温药和之’这一治疗原则是在治疗广义痰饮病时的基本原则，它的含义主要是指使用温性的药物来调和病变。这里的’和’意味着调和、调节，而不是简单的增强或者抑制。

在广义痰饮病的形成过程中，主要是因为肺、脾、肾三脏的阳气虚弱，导致气化不利，水液停聚而成。由于饮为阴邪，遇寒则聚，遇阳则行，得温则化。因此，治疗痰饮需要借助于温性的药物以振奋阳气，开发腑理，通调水道。阳气振奋，既可以温化饮邪，又可以绝痰饮滋生之源。

此外，’温药和之’也强调了对病情的全面考虑和治疗，不仅要针对病因进行治疗，还要考虑到病人的整体状况，使治疗和人体的正常生理机能相协调，达到真正的治疗效果。这种治疗原则体现了中医治疗疾病的整体观念和辨证施治的原则，也是中医学的一种重要治疗思想。

TOSRR answer: The therapeutic principle of ‘harmonizing with the warm medicine’ is a fundamental approach in the treatment of general phlegm and retained fluid diseases. Its essence lies in using medicinal herbs with warm property to harmonize pathological changes. In this context, ‘harmonize’ implies reconciling and regulation, rather than simple enhancement or suppression.

In the formation process of generalized phlegm-damp diseases, the main cause is the weakness of the yang qi in the lungs, spleen, and kidneys, leading to unfavorable qi transformation and the accumulation of fluids. Since retained fluid is characterized as Yin pathogenic factors, it tends to aggregate in the presence of cold and disperse in the presence of warmth, being resolved effectively by warmth. Therefore, the treatment of retained phlegm and fluid involves the use of warm-property herbs to invigorate yang qi, open up sweat pore, and regulate water channels. Invigorating yang qi not only warms and transforms the pathogenic dampness but also eliminates the source of phlegm and retained fluid.

Furthermore, ‘harmonizing with the warm medicine’ emphasizes a comprehensive consideration and treatment of the patient's condition. The focus is not only on addressing the root cause of the disease but also taking into account the overall well-being of the individual. This approach aims to harmonize the treatment with the body's normal physiological functions, achieving genuine therapeutic effects. This therapeutic principle embodies the holistic perspective and the principles of syndrome differentiation and treatment in TCM, representing a significant therapeutic ideology in Chinese medical practice.

TOSRR model recalled 15 SPO-Ts, in which 4 SPO-Ts (6,7,8,11) were closely related to the correct answer and selected by the self-reflection framework（bold font in the table）. The accurate recall helps the model got the right answer.

|  | 召回的SPO-T | Recalled SPO-T |
| --- | --- | --- |
| 1 | Prescription - Warming Interior Formula - Huangqi Guizhi Wuwu Decoction - This paper introduces the therapeutic mechanism of Huangqi Guizhi Wuwu Decoction. This formula is applicable to deficiency of both the nourishing and defensive aspects of qi and blood, where the pathogenic factors of wind-cold invade and assail the blood vessels in the presence of weakness, leading to insufficient nourishment of the muscles and skin, manifesting symptoms such as numbness, mild aversion to cold, and a light red tongue. In contrast to wind-cold arthralgia, this condition, though resembling it, lacks a sense of pain. The pulse is slightly taut and tight, indicative of the characteristic of pathogenic stagnation in the blood vessels with congealed resistance. According to the ‘Su Wen · Counterflow Theory,’ the treatment for this condition should involve supplementing qi, warming the meridians, and promoting blood circulation to alleviate arthralgia. Therefore, Huangqi Guizhi Wuwu Decoction can treat this condition by tonifying qi and blood, and warming the meridians to facilitate unobstructed circulation. | 方剂-温里剂-温经散寒-黄芪桂枝五物汤-本文介绍了黄芪桂枝五物汤的证治机理。该方剂适用于营卫气血不足，风寒之邪乘虚客于血脉，导致肌肤失养，出现麻木、微恶风寒、舌淡等症状。与风痹不同的是，此证虽然类似风痹，但没有疼痛感，脉象微涩兼紧，表现为邪滞血脉、凝涩不通的特征。根据《素问·逆调论》，治疗该证应当益气温经，和血通痹。因此，黄芪桂枝五物汤可以通过补益气血、温经通络的作用来治疗该证。 |
| 2 | Seasonal Febrile Disease - accompanied symptoms and signs - phlegm and restrain fluid - clearing heat, removing phlegm, and resolving congestion- the following passage describes the application of the method of clearing heat, removing phlegm, and resolving congestion in various disease locations. For individuals with phlegm-heat obstructing the lungs, treatment may involve the use of medicines such as melon wilt and tian nan xing. For those with phlegm-heat causing chest congestion, medications like Xiao Xian Xiong Tang could be employed. In cases where phlegm-heat obstructs the orifices, additional herbs such as gallbladder nan xing and da huang may be added to formulas targeting heart-clearing and orifice-opening. Similarly, for individuals with phlegm-heat obstructing the liver meridian, medicines like niu huang and da huang may be included in formulas addressing heat-clearing and wind-dispelling. | 温病-兼夹证-痰饮-清热化痰开结-本节介绍了清热化痰开结法在不同病位的应用。对于痰热雍肺者，可以使用瓜萎、川贝等药物进行治疗；对于痰热结胸者，可以使用小陷胸汤等药物进行治疗；对于痰热闭窍者，可以在清心开窍剂中加入胆南星、大黄等药物；对于痰热阻于肝经者，可以在清热息风剂中加入牛黄、大黄等药物。 |
| 3 | Prescription - Warming Interior Formula – Danggui Si Ni Decoction - the paper explores the therapeutic mechanisms of Danggui Si Ni Decoction in treating conditions such as blood deficiency, cold congealing in the meridians, and impaired blood circulation. The paper emphasizes that the primary causes of this syndrome are internal deficiency of yin-blood and external deficiency of yang-qi. The article also highlights the distinctions between this syndrome and conditions involving the depletion of the heart-kidney yang in the Shaoyin meridian and the extremities' rebellious cold due to internal accumulation of yin-cold. Finally, the paper underscores the treatment approach of warming the meridians, dispelling cold, and nourishing blood to promote meridian circulation. | 方剂-温里剂-温经散寒-当归四逆汤-本文主要讨论了当归四逆汤在治疗营血虚弱、寒凝经脉、血行不利等方面的证治机理。通过许宏的观点，指出阴血内虚和阳气外虚是导致该证的主要原因。文章还提到了该证与少阴心肾阳衰、阴寒内盛之四肢厥逆的不同之处。最后，强调了温经散寒、养血通脉的治疗方法。 |
| 4 | Prescription - Warming Interior Formula – Warming Liver Decoction - The main causes of this syndrome are insufficient liver and kidney function, the presence of cold pathogens in the liver meridian, and stagnation of Qi circulation. Cold, being a Yin pathogen, possesses characteristics of congealing and stagnation. When the functions of the liver and kidneys are weakened, cold pathogens easily invade the liver meridian, leading to impaired circulation of liver meridian Qi and the manifestation of symptoms such as testicular cold pain and lower abdominal discomfort. Treatment of this syndrome should focus on nourishing liver and kidney functions, dispersing cold stagnation, and promoting smooth Qi circulation. | 方剂-温里剂-温经散寒-暖肝煎-肝肾不足、寒客肝脉、气机郁滞是导致本证的主要原因。寒为阴邪，具有凝滞的特性，当肝肾功能不足时，寒邪容易侵袭肝脉，导致肝脉气机不畅，出现睾丸冷痛、小腹疼痛等症状。治疗该证应当补充肝肾功能，散寒凝，促进气机畅通。 |
| 5 | Prescription - Phlegm-expelling Decoction - Warming and Removing Cold Phlegm - San Zi Yang Qin Decoction- This formula is suitable for the elderly with constitutional weakness, spleen deficiency leading to stagnation of dampness from poor digestive function. The accumulated dampness transforms into phlegm, causing obstruction and stagnation of qi, disrupting the ascending and descending functions of the lungs. Symptoms such as coughing and chest tightness may manifest. Concurrently, phlegm-dampness affects the spleen, leading to spleen deficiency and poor digestion, resulting in reduced appetite, a white and greasy tongue coating, and a slippery pulse—typical manifestations of turbid phlegm. The therapeutic principles of this formula involve warming the lungs, transforming phlegm, descending qi, and aiding digestion. | 方剂-祛痰剂-温化寒痰-三子养亲汤-本方适用于年老体虚，脾运不佳导致停食生湿，湿聚成痰，痰浊阻滞，气机雍塞，肺失宣降，出现咳嗽、胸闷等症状；同时痰湿困脾，脾失健运，导致食少难消，舌苔白腻，脉滑，这些都是痰浊的表现。此方的治疗原则是温肺化痰，降气消食。 |
| **6** | **Golden Chamber Synopsis – phlegm and retain fluid – treatment principle – The treatment principles of phlegm and retain fluid diseases involves the use of warm-property herbal medicines.** | **金匮要略-痰饮-治疗原则-痰饮咳嗽病的治疗原则是使用温药** |
| **7** | **Internal medicine of TCM - phlegm and retain fluid diseases - The therapeutic principles for treating phlegm and retained fluid diseases primarily involve the use of warming and transforming methods. Through warming the yang to transform qi, the goal is to facilitate the easy transformation of dampness and the smooth flow of water, allowing the dispersion of phlegm with water. Specific treatment methods vary based on the differentiation of exterior, interior, deficiency, and excess conditions, employing warming, clearing, dispelling, and tonifying techniques accordingly. In cases of abundant phlegm-dampness, the focus is on eliminating the phlegm to address the symptoms; for conditions manifesting on the surface, warming and dispersing with sweating are recommended; for interior conditions, warming and transforming to benefit water metabolism are applied; deficiency conditions require tonification; and excess conditions call for purging methods. In cases involving a combination of excess and deficiency, a comprehensive approach of both dispelling and tonifying is employed. Moreover, if heat is mixed with phlegm, a combination of warming and clearing methods is necessary. In summary, the treatment of phlegm and retained fluid diseases demands flexible therapeutic strategies tailored to the specific condition of the illness.** | **中医内科学-痰饮-痰饮的治疗原则是以温化为主，通过温阳以化气，使饮易化且水易行，饮随水散。具体治法根据表虚实不同，采用温、清、消、补之法。对于水饮雍盛者，应祛饮以治标；邪在表者，应温散发汗；在里者，应温化利水；正虚者则进行补益；邪实者则采用攻破的方法。对于邪实正虚的情况，应综合消补。同时，如果饮热相杂，则需要温清并用。总之，痰饮的治疗需要根据病情采取灵活的治疗方法。** |
| **8** | **Golden Chamber Synopsis – phlegm and retain fluid – The principles for treating phlegm and retained fluid cough are mentioned in the context of the *Golden Chamber Synopsis*. Among them, the principle of ‘harmonizing with warm medical herbs’ is considered a fundamental approach for treating phlegm and retained fluid diseases. Clinical treatment requires specific analysis based on the pathological features. If it is a deficiency syndrome, it is necessary to differentiate between treating the spleen and treating the kidneys. If it is an excess syndrome, therapeutic methods such as promoting Xing, Xiao, Kai, Dao can be employed. ‘行’ (Xing) refers to promoting the flow of qi, ‘消’ (Xiao) means eliminating phlegm, ‘开’ (Kai) indicates opening the yang qi, and ‘导’ (Dao) signifies expelling phlegm through urination and defecation. In summary, when treating phlegm and retained fluid diseases, consideration should be given to the use of warming and tonifying herbs, as well as herbs with actions such as promoting circulation and eliminating dampness.** | **金匮要略-痰饮-金匮要略中提到了治疗痰饮咳嗽病的原则。其中，’温药和之’被认为是治疗痰饮病的基本原则。根据病理特征，临床治疗时需要具体分析。如果是正虚，需要区分治脾和治肾的方法；如果是邪实，则可以采用行、消、并、导等治法。行指行气，消指消除痰液，开指开阳气，导指通过大小便排出痰邪。总之，治疗痰饮咳嗽病时需要考虑温补和行消等药物的使用。** |
| 9 | Golden Chamber Synopsis - phlegm and retain fluid - The main content of this paper focuses on the etiology, pulse manifestations, and treatment of phlegm-damp cough. The article extensively discusses both the general and specific aspects of phlegm-damp, along with the treatment principles and specific therapeutic methods for phlegm-damp diseases. Phlegm-damp is a pathological condition caused by the invasion of dampness evils, with particular emphasis on the involvement of phlegm accumulation. There are four types of phlegm-damp, namely phlegm-damp, hanging-damp, overflowing-damp, and focal-damp. However, this paper primarily addresses the pathological changes of dampness evils lingering in the gastrointestinal tract. The paper proposes the treatment principle of using warming herbs to address phlegm-damp diseases, and introduces specific treatment methods such as warming, inducing sweating, promoting bowel movements, and draining downward, along with adaptive strategies based on symptomatic changes. These insights have significant guiding implications for subsequent clinical practice in the field, especially at the intersection of traditional Chinese medicine and artificial intelligence. | 金匮要略-痰饮-本篇正文是关于痰饮咳嗽病脉证并治的内容。文章重点讨论了痰饮的广义与狭义，以及痰饮病的治疗原则和具体治疗方法。痰饮是由饮邪引起的一种病症，其中支饮是其重点。痰饮包括痰饮、悬饮、溢饮和支饮四种类型，而本篇主要讨论的是饮邪停留在肠胃的病变。文章提出了以温药治疗痰饮病的治则，并介绍了温、汗、利、下等具体的治疗方法以及根据症状变化的应变法则。这对于后世临床实践具有重要的指导意义。 |
| 10 | Internal medicine of TCM – cold - In the treatment of the common cold in TCM, the guiding principle is to resolve from the surface, following the principle outlined in the ‘Su Wen · Yin Yang Ying Xiang Da Lun,’ which states, ‘For those on the skin, induce sweating to expel it.’ For wind-cold patterns, the treatment approach involves using pungent and warm methods to induce sweating. For wind-heat patterns, the treatment involves adopting pungent, cool, and resolving methods. In cases where dampness is mixed with pathogenic factors, the approach is to clear dampness and resolve the surface. In cases of deficiency-type cold, the strategy is to simultaneously resolve the surface and tonify the underlying deficiency. | 中医内科学-感冒-感冒的治疗原则是从表而解，遵循《素问·阴阳应象大论》中的’其在皮者，汗而发之’原则。对于风寒证，应采用辛温发汗的治疗方法；对于风热证，应采用辛凉清解的治疗方法；对于湿邪夹杂的情况，应清湿解表；对于虚体感冒，应同时解表和扶正。 |
| **11** | **Golden Chamber Synopsis – phlegm and retain fluid – This passage discusses the treatment principles for phlegm-dampness syndrome. The formation of phlegm-dampness syndrome is attributed to the weakness of the yang qi in the lungs, spleen, and kidneys, leading to the accumulation of water and fluids. The treatment of phlegm-dampness syndrome involves the use of warming herbs to invigorate yang qi, promote the smooth flow and regulation of water passages, and facilitate the elimination of pathogenic factors. Simultaneously, it is essential to be cautious in the use of warming herbs, avoiding excessive or overly drying and heating effects. The key to treating phlegm-dampness lies in harmonizing the body's yang qi, which is the fundamental approach to addressing the root cause.** | **金匮要略-痰饮-本条论述了治疗痰饮病的治疗原则。痰饮病的形成是由于肺脾肾三脏阳气虚弱，导致水液停聚。治疗痰饮病需要借助于温药来振奋阳气，开发通调水道，以疏通祛邪。同时，治疗痰饮需要注意温药的使用不可过度，也不可过于燥热。治疗痰饮的关键是调和人体阳气，这是治本之法。** |
| 12 | Internal medicine of TCM - phlegm and retain fluid diseases - Phlegm-dampness refers to the abnormal distribution and metabolism of body fluids, resulting in the main symptoms of accumulation in certain areas. It encompasses four categories: phlegm-dampness, suspended dampness, overflowing dampness, and obstructive dampness. Clinical manifestations of phlegm-dampness are diverse, including chronic bronchitis, bronchial asthma, exudative pleurisy, chronic gastritis, heart failure, and edema associated with nephritis. The formation and development of the theory of phlegm-dampness have laid a theoretical foundation. Zhang Zhongjing, in his work ‘Jin Kui Yao Lue,’ was the first to classify phlegm-dampness broadly into four categories and proposed corresponding treatment principles and prescriptions. From the Sui and Tang dynasties to the Jin and Yuan dynasties, a phlegm-dampness syndrome system gradually formed with phlegm at its core. After the Song dynasty, medical scholars conducted more in-depth research and summarization of the theory and treatment methods of phlegm-dampness. | 中医内科学-痰饮-痰饮是指体内水液输布、运化失常，停积于某些部位为主症的疾病。痰饮包括痰饮、悬饮、溢饮、支饮四类。痰饮的临床病种广泛，表现复杂，包括慢性支气管炎、支气管哮喘、渗出性胸膜炎、慢性胃炎、心力衰竭、肾炎水肿等。痰饮学说的形成和发展奠定了理论基础。张仲景在《金匮要略》中首次将广义痰饮分为四类，并提出了相应的治疗原则和方剂。隋唐至金元时期逐渐形成了以广义的痰为核心的痰饮病证体系。宋代以后，医家对痰饮的理论和治疗方法有了更深入的研究和总结。 |
| 13 | Internal medicine of TCM - phlegm and retain fluid diseases - This paper introduces and discusses the treatment principles and essence of phlegm-dampness syndrome based on two classical Chinese medical texts, *Synopsis of Golden Chamber*  and *Water, Dampness, Phlegm, Fluids*. In  *Synopsis of Golden Chamber,* the primary approach to treating phlegm-dampness syndrome is through the use of warm-tonifying herbs. On the other hand, *Water, Dampness, Phlegm, Fluids* analyzes the essence and treatment principles of phlegm-dampness. Additionally, the literature *On the essence of phlegm and drink* provides further insights into the essence of phlegm-dampness. Through the introduction and analysis of these texts, a better understanding and treatment approach for phlegm-dampness syndrome can be achieved in the intersection of traditional Chinese medicine and artificial intelligence. | 中医内科学-痰饮-本文介绍了《金匮要略方论》和《水、湿、痰、饮》两篇文献对于痰饮证的治疗原则和实质的探讨。其中，《金匮要略方论》认为痰饮证应该以温药治疗为主，《水、湿、痰、饮》则对痰饮的实质和治疗原则进行了分析。另外，还有一篇文献《论痰饮实质》对痰饮的实质进行了论述。通过这些文献的推介和分析，可以更好地理解和治疗痰饮证。 |
| 14 | *Treatise of Febrile Disease*s -The name ‘*Treatise of Febrile Disease*s’ is derived from the term 'Shanghan,' and its meaning has both broad and narrow interpretations. In a broad sense, Shanghan encompasses all febrile diseases caused by external pathogens, while in a narrow sense, it specifically refers to diseases arising from the invasion of wind-cold factors. The text primarily discusses Shanghan, Zhongfeng, Wenbing(seasonal febrile diseases), and others, falling within the category of broad Shanghan. It is crucial to emphasize that the concept of 'Shanghan' as discussed in ' *Treatise of Febrile Disease*s' is entirely different from the Western medical understanding of 'typhoid fever.'‘ | 伤寒论-《伤寒论》以伤寒命名，其含义广义和狭义有所区分。广义伤寒是指一切外感热病的总称，而狭义伤寒则是指外感风寒，感而即发的疾病。书中主要讨论了伤寒、中风、温病等，属于广义伤寒的范畴。需要强调的是，《伤寒论》所论的伤寒病与西医学中的’伤寒’含义完全不同。 |
| 15 | Golden Chamber Synopsis – phlegm and retain fluid – This paper primarily discusses diseases related to phlegm-fluid retention and cough, emphasizing the significance of phlegm-fluid retention. The formation of phlegm-fluid retention is associated with the spleen, lungs, and kidneys, with a particularly close relationship to the spleen. Phlegm-fluid retention can be broadly and narrowly classified. Broadly, it includes four types: phlegm-fluid retention, suspended fluid, overflowing fluid, and branchial fluid. Narrowly, phlegm-fluid retention specifically refers to lesions in the gastrointestinal tract.  The affected areas of phlegm-fluid retention are mainly in the gastrointestinal tract, subcostal region, thoracic diaphragm, and the interstitial tissues of the limbs, and it can also impact the five viscera. The classification of the four types of fluids is based on the location of water retention and the main symptoms. Different prescriptions can be used interchangeably. The principle of treating phlegm-fluid retention is primarily based on the use of warming drugs, and the treatment methods should also be adapted according to conditions such as deficiency or excess, cold or heat. | 金匮要略-痰饮-本篇主要论述痰饮和咳嗽的疾病，并强调了痰饮的重要性。痰饮的形成与脾、肺、肾有关，尤其是与脾的关系密切。痰饮可以广义和狭义来分类，广义痰饮包括痰饮、悬饮、溢饮和支饮四种，而狭义痰饮仅指胃肠的病变。痰饮的病变部位主要在胃肠、胁下、胸隔和肢体肌肤之间，并且可以影响五脏。四饮是根据水饮停聚的部位和主要症状进行分类的，同时不同的方剂可以互相通用。治疗痰饮的原则是以温药和之为主，同时还需根据虚实、寒热等情况采用相应的治疗方法。对于支饮服用小青龙汤后的变证处理，是辨证论治的示范。本篇中涉及的方剂在其他篇和《伤寒论》中也有出现，学习时需要相互对勘，加深理解，以便灵活应用。 |
